# A Human Oral Fluid Assay for *D*- and *L-* Isomer Detection of Amphetamine and Methamphetamine Using Liquid-Liquid Extraction

**DOI:** 10.1155/2022/4819599

**Published:** 2022-12-02

**Authors:** Brian Robbins, Rob E. Carpenter, Mary Long, Jacob Perry

**Affiliations:** ^1^Department of Research, Advanta Genetics, 10935 CR 159 Tyler, Texas 75703, USA; ^2^University of Texas at Tyler, 3900 University Boulevard, Tyler, Texas 75799, USA; ^3^University of Alabama at Birmingham, 1720 University Blvd, Birmingham, AL 35294, USA; ^4^University of Miami Miller School of Medicine, 1600 NW 10th Ave, Miami, FL 33136, USA

## Abstract

Medical providers are increasingly confronted with clinical decision-making that involves (meth)amphetamines. And clinical laboratories need a sensitive, efficient assay for routine assessment of *D*- and *L*-isomers to determine the probable source of these potentially illicit analytes. This paper presents a validated method of *D*- and *L*-isomer detection in human oral fluid from an extract used for determination of a large oral fluid assay (63 analytes) on an older AB SCIEX 4000 instrument. Taken from the positive extract, *D*- and *L*-analytes were added. The method for extraction included addition of internal standard and a 2-step liquid-liquid extraction and dry-down step to concentrate and clean the samples. The samples were suspended in 50% MeOH in water, diluted with mobile phase, with separation and detection accomplished using LC-MS/MS to determine analyte concentration. Once samples were confirmed positive for (meth)amphetamine from the large oral fluid assay, they were further examined for the enantiomeric forms with 50 *μ*l aliquots of the standards and samples of interest combined with 450 *μ*l of *D-* and *L-*assay mobile phase, then analyzed using chiral column separation, and LC-MS/MS detection with standard curve spanning the range from 2.5 to 1000 ng/mL. The result is a sensitive and accurate detection of *D-* and *L-*isomers of amphetamine and methamphetamine in human oral fluid performed on an older model mass spectrometer (AB SCIEX 4000). The novelty of this assay is twofold (a) the 2-step liquid-liquid extraction and dry-down step to concentrate and clean the samples, and (b) its adoption characteristics as a reflex test from a large ODT panel without the need to invest in newer or expensive LC-MS/MS instruments. Finally, this assay also has potential to add a valuable option to high-throughput laboratories seeking a *D-* and *L-*testing alternative to urine drug testing methods.

## 1. Introduction

Amphetamines and methamphetamine are popular illicit drugs of abuse for their stimulation of the central nervous system. These central nervous system (CNS) stimulants exist as two enantiomeric forms, dexter (*D-*) or laevus (*L-*), which produce radically different effects on the human system. Notwithstanding the potential use disorder from the heighted dopamine response that *D-* (meth)amphetamine asserts [1, 2], the *L-*form is quite common and is an effective vasoconstrictor used in the over-the-counter formulation of Vicks® VapoInhaler™. Moreover, the *D-*form can be used therapeutically as a treatment for overeating disorders, narcolepsy, and attention deficit disorder, but produces the sought-after habit-forming central nervous system (CNS) effects that can be abused and long term neurotoxicity [2–4]. Thus, medical providers are increasingly confronted with clinical decision-making that involves (meth)amphetamines—including illicit use.

Routine assessment for noncompliance or nonmedical use of (meth)amphetamine is frequently accomplished through urine drug testing (UDT) based on risk of drug misuse, abuse, and diversion. Although UDT is considered the common practice for detecting scheduled drug compliance or illicit use, often medical providers are unable to procure a urine sample for various reasons. In this case, oral fluid drug testing (ODT) can serve as an effective alternative to UDT [5]. ODT is increasingly emerging as an alternative biological matrix for detecting drugs and monitoring patient medication compliance [6–8]. Although the detection window for oral fluid is small compared to matrices like urine, hair, or sweet, in certain clinical situations—like immediate detection of recent marijuana use—oral fluid shows to be more beneficial over UDT [9, 10]. The matrix allows for easy collection, but attention to recovery, stability, and dilutions issues of some collection devices should be given consideration for pharmacokinetic studies [10, 11].

Although ODT opioid assays that use dilute-and-shoot methods with little sample manipulation have been developed and validated on AB SCIEX 4500 instruments with excellent calibration ranges (2.5–1,000 ng/mL) [12] robust ODT assays that also quantify the *D-* and *L-*isomers of (meth)amphetamine are less common. Notably, there are several methods available for determining *D-* and *L-* (meth)amphetamine including immunoassays which have been designed to react with one or the other but often have problems with cross-reactivity. Gas chromatography–mass spectrometry (GC-MS) can also be used but often requires an extra step requiring the synthesis of derivatives resulting in potential purity issues and errors in concentration estimation. Other methodologies including some liquid chromatography with tandem mass spectrometry (LC-MS/MS) methods also require purification steps with solid phase extraction cartridges involving additional equipment and the cartridges themselves adding extra cost per sample. Accordingly, there is a need for developing an efficient method to detect enantiomeric forms of *D-* and *L-* (meth)amphetamine, especially in small resource-limited laboratories without the capacity to invest in newer LC-MS/MS instruments.

In this paper, we offer a novel approach for a fast, accurate, and applicable method to quantify the *D-* and *L-*isomers of (meth)amphetamine in human oral fluid specimens using liquid-liquid extraction and LC-MS/MS with an older model AB SCIEX 4000 instrument as an extension of larger ODT assay. A preprint version of this assay paper has been published [13]. Each patient sample was initially analyzed for 63 targeted analytes using LC-MS/MS with the same extracts injected a second time using a delta-9 tetrahydrocannabinol (THC)-specific (ES) negative assay to capture the THC. Then, for samples that exhibited a positive confirmation result for (meth)amphetamine, an additional sample was taken from the previously extracted specimen. This sample was then analyzed with a newly developed assay designed specifically to assess the *D-* and *L-*isomer status to define nonillicit versus illicit etiology. The assay development and validation offered here is for the benefit of high-throughput laboratories seeking novel solutions for a quantitative *D-* and *L-*isomer test from oral fluid with fast and accurate chemical analysis using less expensive older model AB SCIEX instruments. Although other methodologies may be more economic for this purpose, this assay provides small resource-limited laboratories with older model AB SCIEX 4000 instruments to operationalize discovery of enantiomeric forms of *D-* and *L-* (meth)amphetamine without incurring additional equipment expensive.

## 2. Methods and Materials

### 2.1. Reagents and Standards

All analyte stock solutions at 1 mg/mL concentrations and deuterated internal standards at 100 *μ*g/mL were purchased from Cerilliant Corporation (Round Rock, TX, USA). All organic solvents including methanol, acetonitrile, formic acid (88%), dichloromethane, 2 propanol, and ethyl acetate were obtained from Fisher Scientific (Pittsburgh, PA, USA). Because experiments have demonstrated considerable variation in recovery of (meth)amphetamine from various oral fluid collections devices [14], the Quantisal™ oral fluid collection device was used based upon its exhibited recovery of (meth)amphetamine shown to exceeded 93%, and the device has seen good recovery in liquid-liquid extraction techniques [15, 16]. The Quantisal™ oral fluid collection device and extraction buffer were obtained from Immunalysis Corporation (Pomona, CA, USA).

### 2.2. Mobile Phase and Extraction Solutions

A *D-* and *L-*mobile phase (MPDL) solution was created by adding ∼993.2 mL of methanol to a 1 L bottle. Then using a 1 mL pipettor, 5 mL of type I water, 1.5 mL of acetic acid, and 0.3 mL of ammonium hydroxide were added. This solution can be kept at room temperature for up to 1 year. Extraction solution 1 (ES1) was created with 50% dichloromethane and 50% 2-propanol by using a graduated cylinder under a fume hood. Equal volumes of dichloromethane and 2-propanol were added to a clean reagent bottle which was capped and mixed well. Extraction solution 2 (ES2) was created with 50% dichloromethane and 50% ethyl acetate by using a graduated cylinder under a fume hood. Equal volumes of dichloromethane and ethyl acetate were added to a clean reagent bottle which was capped and mixed well.

### 2.3. Standard Preparation

An 8000 ng/mL stock solution was made by combining analyte stock controls and diluting it with MPA. In contrast, *D-* and *L-*amphetamine and methamphetamine were added in an amount to make a 4000 ng/mL stock of each isomer so that when combined they would produce an 8000 ng/mL solution of total amphetamine and methamphetamine. This means that the range of the *D*-and *L* standard curve (SC) is from 2.5 to 1000 ng/mL (half the concentration). The resulting stock standard was diluted with mobile phase A (MPA) to produce the SC. Concentrations were 8000 (undiluted), 4000, 2000, 1000 400, 200, 100, 40, 20, 10, 4, and 2 ng/mL. These solutions were stored at the concentrations above. They underwent a dilution during the assay (1 part standard to 3 parts mobile phase and THC standard) to achieve the concentration desired in sample analysis with oral fluid (saliva). The standards and quality control (QC) were diluted (0.5 mL) with 1.5 mL of extraction buffer. This approximates the condition seen with saliva after collection with the Quantisal™ oral fluid sample collection device. The final concentration in the 0.5 mL sample SC included the following points: 2000, 1000, 500, 250, 100, 50, 25, 10, 5, 2.5, 1, and 0.5 ng/mL.

The assay QCs were made similarly; first making a 7200 ng/mL spiking solution in MPA then diluting to 3200, 2400, 300, 60, 12, and 2 ng/mL. The *D-* and *L-*amphetamine and methamphetamine QCs were made at half concentrations. Final concentrations of each QC were 1800, 800, 600, 75, 15, 3, and 0.5 ng/mL, after the 1 : 4 dilution with MPA and THC QC same as the SC points noted above.

The internal standard working solution (ISWS) for the large oral fluid assay and the *D-* and *L-*assay was made by filling a 100 mL graduated cylinder to the 50 mL mark with 10% methanol in water and adding 250 *μ*L of each of the internal standards listed above. The volume was brought to 100 mL with additional 10% methanol producing a concentration of 250 ng/mL.

### 2.4. Instrumentation

The liquid chromatography components of the LC-MS/MS system consisted of a model CBM-20A controller, 2 model Prominence LC-20AD pumps, a model DGU-20A5 degasser, and a model SIL-20AC autosampler all obtained from (Shimadzu, Columbia MD, USA, based in Kyoto, Japan). The mass spectrometer used was a SCIEX API 4000 and the acquisition software was Analyst, v 1.5.2, build 5704 (Framingham, MA, USA). Nitrogen was obtained using a Peak ABN2ZA gas generator (Peak Scientific, Billerica, MA, USA). Reagents were weighed on a Mettler Toledo MX5 analytical micro balance (Fisher Scientific, Pittsburgh, PA, USA). Samples were dried on a TurboVap® LV (Uppsala, Sweden). Samples were vortexed on a Fisherbrand 120 multitube vortex. The analytical column was an Astec CHIROBIOTIC® V2 5.0 *μ*m (2.1 mm × 25 cm column) Catalog # 15020AST SUPLECO®, (Bellefonte, PA, USA).

### 2.5. Analyte Optimization

Individual analytes and internal standards were optimized by using T-infusion with 50% B mobile phase and tuning for declustering potential (DP), entrance potential (EP), collision energy (CE), and exit potential (CXP) at a flow rate of 0.7 mL/min. The two most abundant fragments were selected for monitoring using multiple reaction monitoring (MRM).

### 2.6. Sample Preparation and Procedures

Samples were collected using the Quantisal™ oral fluid collection device. The manufacturers collections instructions were followed (https://immunalysis.com/products/oral-fluid/quantisal/). The samples, standards, and QC were extracted using two liquid-liquid extractions with 1 : 1 DCM : IPA and 1 : 1 DCM : EtAc. They were combined, dried, reconstituted with 50% MeOH water, and combined with mobile phase for separation of the analytes. Sample preparation for *D-* and *L-*analysis by LC-MS/MS involved transferring 50 *μ*L of the already extracted standards, QC, and any samples of interest to a new plate. Then 450 *μ*L of MPDL was added to each well and mixed with a multichannel pipette, the plate was covered with a plate mat and analyzed for the *D-* and *L-*isomers of amphetamine and methamphetamine using the listed chiral column. The LC-MS/MS conditions and separation parameters are presented in Table 1.

## 3. Method Validation Procedures

The assay was developed as an extension of a larger 63 analyte assay whereby samples that were confirmed positive for (meth)amphetamine could be reflex tested to identify nonillicit versus illicit etiology. The assay was initially optimized for extraction of all the required analytes in the larger panel. Samples were spiked with a combination of all the drugs of study and multiple solvent systems and single and two step processes were evaluated including the combinations finally selected; these were dichloromethane plus isopropyl alcohol for solution 1 and dichloromethane plus ethyl acetate for solution 2 as these appeared to be the most broad-spectrum solvents for extraction of the different analytes tested—including methamphetamine and its metabolite amphetamine, and analytes with different chemical solubility characteristics such as meprobamate. Moreover, the *D-* and *L-*isomer component of this assay was adapted from a dilute and shoot method for analysis of urine samples and was modified to the back end of the oral fluid assay after extraction. Importantly, it was found during development that the elimination of phentermine was achieved by both chromatographic separation and elimination by careful selection of the secondary transition.

### 3.1. Matrix Lot-to-Lot Comparison

Individual lots of human matrix (saliva) differ according to a person's overall health and hydration status [17]. A single lot of oral fluid is not enough to demonstrate the ruggedness of the assay system when such variability in the matrix exists [18]. Due to this, and in accordance with the current College of American Pathologists (CAP) standards, a minimum of 10 lots of human matrix were collected from drug-free donors. These oral fluid samples were spiked at a low-level concentration with each analyte. These samples were prepared, extracted, and run as described above. The responses were calculated and the analyte to internal standard (IS) ratio and %CV is shown in Table 2.

### 3.2. Analytical Measurement Range

The analytical measurement range (AMR) of the assay refers to the concentration range that the assay is validated within and is determined by running a series of calibration curve standards covering a concentration range that encompass the concentration of analyte expected to find in patient samples [19]. The limits of the AMR were bounded by the lower limit of quantitation (LLOQ) and the upper limit of quantitation (ULOQ). The dynamic range may be described by a linear or quadratic fit [19, 20]. Calibration curves were created using a minimum of six nonzero calibration points. To be accepted as the AMR, all points describing the calibration curve must pass within ±20% of the nominal concentration [19]. Furthermore, the correlation coefficient (*R*^2^) for the calibration curve must be ≥ 0.99, or *R* should be ≥ 0.98 to be acceptable [21, 22].

### 3.3. Sensitivity

The sensitivity of the assay system refers to the ability to reliably produce a signal throughout the entire calibration range, but specifically at the low-end of the calibration curve (the lower limit of quantitation, LLOQ) [23]. In hyphenated mass spectrometry assays, a signal that produces a signal to noise ratio (S/N) of ≥10 is considered valid for the LLOQ of an assay system [24]. Further, an *S*/*N* ratio of ≥5 is considered clear enough for the limit of detection. The sensitivity of the assay system was tested by injecting 6 replicates of the LLOQ over 3 days and evaluating the resulting analytical determinations. Standard acceptance criteria of ±20% of nominal concentration apply.

### 3.4. Intraday Precision and Accuracy

Intraday precision and accuracy were determined using six replicates of each of three QC sample determinations and LLOQ from across at least three validation runs. Concentrations of the QC samples ranged across the curve, with the low QC set at approximately 3 times the LLOQ or less, the mid QC near the mid-range of the linear range of the curve, and the high QC set at 80–90% of the ULOQ. Percent accuracy and precision were determined for each individual measurement. To be accepted, the precision and accuracy for the replicate determinations must be ≤ 20% at each level.

### 3.5. Interday Precision and Accuracy

Interday precision and accuracy were determined using all replicates of each of three quality control (QC low, QC mid, and QC high) and LLOQ sample determinations from the analytical runs performed on 3 separate days. Concentrations of the QC samples ranged across the curve, with the low QC set around 3 times the LLOQ, the mid QC near the middle of the linear range, and the high QC set at 80–90% of the ULOQ. To be accepted, the precision and accuracy for the replicate determinations must be ≤ 20% at each level.

### 3.6. Exogenous Interfering Substances

Drugs that are known or suspected of interfering with similar bioanalytical systems should be evaluated to ensure that they do not suppress ionization or cause false-positive results for a given analyte [25, 26]. The following medications were evaluated: over-the-counter mix, acetaminophen, ibuprofen, pseudoephedrine, caffeine, and naproxen. The following individual analytes were also tested: salicylic acid, phenylephrine, phentermine, diphenhydramine, and dextromethorphan. A high concentration of the possible interfering drug (typically 2,000 ng/mL or greater) was spiked into a low QC sample (15–75 ng/mL low QC). Acceptance criteria for a substance to be deemed as noninterfering is that the quantitated value for the low QC should be within ±20% of the nominal value [27]. Furthermore, the spiked substance should not cause a false-positive or a false-negative result.

### 3.7. Partial Volumes and Dilutions

A spiked solution was created at a concentration above the ULOQ, in this case, 4000 ng/mL. The sample was run at discrete dilutions of 1 : 5, 1 : 10, 1 : 20, and 1 : 50. Concentration determinations for all dilutions should be within ±20% of the nominal value following correction for the dilution factor [27, 28]. More recent literature suggests that the signal to noise ratio of both the quantification trace and the qualifying ion trace be 3–10 [29]. On occasion, an analyte will not have a quantifying ion that passes this criterion while still permitting the quantification trace to remain in a meaningful range. These instances should be documented in the laboratory standard operating procedure or validation report.

### 3.8. Carryover

Carryover is the presence of an analyte in a blank injection following a positive injection, resulting in a false-positive sample [30]. The injection needle should be washed in-between samples with a needle wash solution that is intended to remove contamination from the surface of the needle. The efficiency of this process is monitored during validation by assessing carryover in the following manner. Samples are injected in the following sequence: high QC, wash, high QC, wash, high QC, wash. Peak areas are integrated for both the analyte and internal standard. Peak area in the wash solutions should be 0.1% or less of that found in the high QC standard. In addition, the mean of the peak area in the three wash solutions following the high QC replicates should be less than 20% of the LLOQ being used for the assay [30].

## 4. Results

### 4.1. Interday Average back Calculated Calibration Standards

Each validation run contained calibration standards with theoretical concentrations of 1, 2.5, 5, 10, 25, 50, 100, 500, 1000, and 2000 ng/mL of each of the analytes with an additional negative run at 0.5 ng/mL.  Table 3 shows the range of standard curves of the combined amphetamine and the individual *D*- and *L*-analytes and the correlation information. *D*- and *L*-curve concentrations were half the above concentration ranging from 0.25 (neg) to 1000 ng/mL. Mean *R* values were all at least 0.99 indicating good fit to the data.

### 4.2. Accuracy and Precision, LLOQ

Six replicates of each validation level were run on at least 3 days. The theoretical concentrations were 1, 5, or 25 for LLOQ on the combined concentrations, 3, 15, or 75 for QC low, 600 ng/mL for QC mid, and 800 or 1800 for the QC high values. The *D-* and *L-*assay individually had an LLOQ of 2.5 ng/mL with a QC low of 7.5 ng/mL, a QC mid of 300 ng/mL, and a high QC of 900 ng/mL. Tables 4–6 indicate mean, interassay, and intraassay statistic variability were all below 20%.

### 4.3. Partial Volumes Accuracy and Precision

An MPA surrogate sample was prepared at 4000 ng/mL. To determine the concentration of this sample, a dilution must be made so the final concentration would be less than 2000 ng/mL to get it in the measurement range of the assay. Three replicates of four dilutions were made and tested: (1) 1 : 5 target 400 ng/mL; (2) 1 : 10 with a target of 200 ng/mL; (3) 1 : 20 with a target of 100 ng/mL; and (4) 1 : 50 with a target of 40 ng/mL. The results shown in Table 7 indicate that all analytes can be diluted at all levels.

### 4.4. Room Temperature, Refrigerator, and Freezer Stability

Samples with concentrations of 75, 800, or 1800 ng/mL were prepared in triplicate. One set was kept at room temperature overnight (RT), a second set was kept in the refrigerator overnight (RF), and a third set was kept in the freezer overnight (FZ). These validation samples were then run and compared to a triplicate preparation of QC samples that had been analyzed as normal. All results show less than 20% deviation from expected (Table 8).

### 4.5. Freeze-Thaw (FT) Stability

Validation samples with concentrations of 75, 800, or 1800 ng/mL were frozen at −20°C and thawed in sequence with samples taken after each freeze-thaw cycle for a maximum of three cycles. These validation samples were analyzed in triplicate and compared to a triplicate preparation of validation samples that had not been subjected to this freeze-thaw cycle. The experimental results showed all meeting acceptance criteria.

### 4.6. Extracted Sample Stability

A stability experiment was performed where samples were stored in the instrument (3 day) or refrigerator (7 day) and reinjected after 3 and 7 days. All samples were within 20% of the initial results.

### 4.7. Stability in Matrix

A series of triplicate samples were analyzed over 7 days for stability at room temperature, 4°C and −20°C. The results indicated that all analytes were stable for at least 7 days refrigerated and frozen. The analytes were stable at room temperature for 24 hours.

### 4.8. Matrix Recovery/Matrix Effects

Table 9 indicates the effect of 10 different matrix lots tested by using a series of 7.5 ng/mL samples prepared in water, MPA, and 10 different matrices. The results were acceptable with less than 20% CV across oral fluid, water, and MPA meeting acceptance criteria. This is likely due to dilution in 1.5 mL Quantisal™ extraction buffer before extraction.

### 4.9. Selectivity

Multiple drugs that might have a potential for interfering with the assay analytes were run in the assay. Samples of 500 *μ*L of 7.5 ng/mL QC were placed in a series of tubes to be run in triplicate. To the first set, 50 *μ*L of MeOH was added to act as the control. To the remaining tubes, 50 *μ*L of sample containing dextromethorphan, diphenhydramine, phenylephrine, salicylic acid, or combo (includes acetaminophen, caffeine, chlorpheniramine, ibuprofen, naproxen, and pseudoephedrine). These solutions were obtained from Cerilliant and were at a concentration of 1 mg/mL each except for the over-the-counter mix which was 100 *μ*g/mL. Each solution was diluted to 20 *μ*g/mL in methanol and this solution was used to spike samples as indicated above. All samples met the accepted criteria and are diplayed in Table 10 and Table 11.

## 5. Discussion

**Figure 1:**
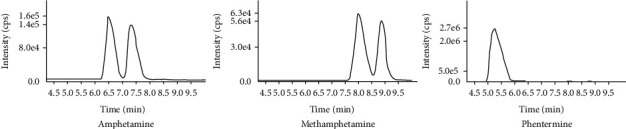
MRM chromatograms of amphetamine, methamphetamine, and phentermine.

The determination of prescription medications and illicit substances is needed for medical compliance [31]. And human oral fluid is one of the most noninvasive and easily observed sample collection methods. It provides a relatively simple and reliable means of sample collection coupled with a reduced chance of sample adulteration. Oral fluid also provides a viable alternative for measurement in patients that cannot provide an adequate urine sample volume such as catheterized patients. The drawbacks of the oral fluid assay are that it has a shorter detection window and requires a more sensitive assay. Accordingly, this paper demonstrates a developed and validated cost-effective means of analysis using older, less sensitive instruments (API SCIEX 4000) by using a 2 step liquid-liquid extraction method and concentration of the samples with a nitrogen dry-down and a resuspension step. The method of development of this *D-* and *L-*assay was validated in accordance with the United States Food and Drug Administration and the College of American Pathologists guidelines [30] with an LLOQ of 2.5 ng/mL and ULOQ of 1000 ng/mL. The most important aspect of this assay was its specificity. It has the ability to reliably and definitively differentiate between the isomeric forms of methamphetamine and its metabolite amphetamine, as well as common decongestants and weight loss medication. Phentermine in particular is a positional isomer of methamphetamine that laboratories need to ensure does not interfere in (meth)amphetamine confirmation. As a positional isomer, it shares a molecular weight and fragment pattern nearly indiscernible from methamphetamine by many LC-MS/MS methods. This assay showed good chromatographic separation of methamphetamine, amphetamine, and phentermine (Figure 1), permitting clear differentiation between these positional isomers.

## 6. Conclusion

The development and validation of an ODT assay designed specifically to assess the *D-* and *L-*isomer status of (meth)amphetamine to define nonillicit versus illicit etiology is presented. The novelty of this assay is twofold (a) the 2-step liquid-liquid extraction and dry-down step to concentrate and clean the samples, and (b) its adoption characteristics as a reflex test from a large ODT panel without the need to invest in newer or expensive LC-MS/MS instruments. The assay is quite sensitive with a cutoff of 2.5 ng/mL and has good precision and accuracy. Although other methodologies may be more economic for enantiomeric discovery, this assay provides small resource-limited laboratories with existing older model AB SCIEX 4000 instruments an effective way to operationalize the identification of *D-* and *L-*forms of (meth)amphetamine without incurring additional equipment expense. Finally, this assay also has potential to add a valuable option to high-throughput laboratories seeking a robust testing alternative to UDT methods.

## Figures and Tables

**Table 1:** LC-MS/MS conditions for the three assays on a single oral fluid sample.

	*D-* and *L-*
Scan type	MRM
Ion source	Turbo spray
Probe position	*X* = 5.00, *Y* = 5.2
Polarity	Positive
Run duration	11 min
Settling time (msec)	0
Pause time (msec)	7.007 msec
Curtain gas	35
CAD gas	4
ISV (V)	5000
Temperature (°C)	500
Ion source gas 1 (GS 1)	50
Ion source gas 2 (GS 2)	50
Q1/Q3 resolution:	unit/unit
CEM (V)	2600
Inlet settings	
Analytical column	Supelco Astek Chirobiotic V 250 × 2.1 mm, 5 *μ*m
Guard cartridge	None
Sample temperature	15 ± 5.0°C
Column temperature	30.0 ± 5.0°C
Mobile phase A	Water : acetic acid : ammonium hydroxide : Methanol 5 : 1 : 0.3 : 993.5
Mobile phase B	N/A
Needle rinse	Water : acetic acid : ammonium hydroxide : Methanol 5 : 1 : 0.3 : 993.5
Flow rate	0.3 mL/min
Injection volume	10 *μ*L
Run time	11 min

*Note.* collision gas (CAD), ion source voltage (ISV), channel electron multiplier (CEM).

**Table 2:** Matrix effects 10 different lots of oral fluid were fortified with QC material to a concentration of 7.5 ng/mL and the %CV determined of the analyte/IS area ratio.

	Matrix comparison
Drug/Metabolite	%CV analyte/IS ratio
Amphetamine^*∗*^	3.39
Methamphetamine^*∗*^	4.60
*D-*amphetamine	1.59
*L-*amphetamine	2.35
*D-*methamphetamine	7.54
*L-*methamphetamine	3.09

^*∗*^Indicates combined *D-* and *L-*.

**Table 3:** Statistical analysis for each analyte standard curve over three assays.

Drug/Metabolite	Curve range (ng/mL)	Mean R	RSD	Mean slope	SD slope	N	Fit
Amphetamine^*∗*^	5–1000	0.9990	0.0006	0.0594	0.0037	3.0000	Quadratic
Methamphetamine^*∗*^	5–2000	0.9999	0.0001	0.0104	0.0004	3.0000	Quadratic
*D-*amphetamine	2.5–1000	0.9994	0.0003	0.0104	0.0010	3.0000	Quadratic
*L-*amphetamine	2.5–1000	0.9993	0.0006	0.0105	0.0010	3.0000	Quadratic
*D-*methamphetamine	2.5–1000	0.9995	0.0008	0.0218	0.0021	3.0000	Quadratic
*L-*methamphetamine	2.5–1000	0.9985	0.0021	0.0246	0.0031	3.0000	Quadratic

^*∗*^Indicates combined *D-* and *L-*.

**Table 4:** Interassay mean and standard deviation (SD) of validation samples.

Drug/Metabolite	LLOQ (ng/mL)	LQC (ng/mL)	MQC (ng/mL)	HQC (ng/mL)
	Mean ± SD	Mean ± SD	Mean ± SD	Mean ± SD
Amphetamine^*∗*^	5.4 ± 0.5	16.7 ± 0.7	581.5 ± 24.6	802.4 ± 64.4
Methamphetamine^*∗*^	5.1 ± 0.4	15.1 ± 0.5	585.6 ± 25.9	1837 ± 85.7
*D-*amphetamine	2.8 ± 0.1	8.3 ± 0.4	295.5 ± 14.8	940.3 ± 33.4
*L-*amphetamine	2.8 ± 0.1	8.4 ± 0.3	294.5 ± 6.1	874.7 ± 25.7
*D-*methamphetamine	2.6 ± 0.1	7.6 ± 0.3	300.6 ± 12.1	999.5 ± 48.9
*L-*methamphetamine	2.6 ± 0.1	7.7 ± 0.4	301.6 ± 16.1	1013.9 ± 51.4

^*∗*^Indicates combined *D-* and *L-*.

**Table 5:** Interassay precision and accuracy: precision and accuracy over 3 days with replicates of 6 for a total of 18 samples.

Drug/Metabolite	LLOQ		LQC		MQC		HQC	
	%CV	%E	%CV	%E	%CV	%E	%CV	%E
Amphetamine^*∗*^	9.25	7.37	4.31	11.13	4.22	−3.09	8.02	0.30
Methamphetamine^*∗*^	8.52	2.81	3.34	0.69	4.42	−2.40	4.66	2.08
*D-*amphetamine	3.95	13.09	4.54	10.70	1.80	−1.52	3.55	4.47
*L-*amphetamine	4.12	10.00	3.04	11.52	2.07	−1.85	2.94	−2.81
*D-*methamphetamine	4.44	3.17	3.98	1.30	4.04	0.21	4.89	11.05
*L-*methamphetamine	5.54	3.21	4.98	2.33	5.32	0.52	5.07	12.66

^*∗*^Indicates combined *D-* and *L-*.

**Table 6:** Intraassay precision and accuracy: precision and accuracy over 3 days with replicates of 6 for each day.

Drug/Metabolite	LLOQ				LQC				MQC				HQC			
	%CV		%E		%CV		%E		%CV		%E		%CV		%E	
	MIN	MAX	MIN	MAX	MIN	MAX	MIN	MAX	MIN	MAX	MIN	MAX	MIN	MAX	MIN	MAX
Amphetamine^*∗*^	2.28	10.19	−2.20	13.20	2.72	5.62	9.07	12.43	2.06	5.57	−5.07	−0.91	3.46	8.89	−5.00	6.59
Methamphetamine^*∗*^	4.84	9.89	−2.60	7.53	1.71	4.41	−0.02	1.66	1.71	2.92	−6.31	2.48	2.10	4.41	−2.22	6.50
*D-*amphetamine	1.38	3.51	8.44	15.79	1.14	1.40	4.38	15.69	0.69	1.73	−2.89	-0.02	1.46	2.99	0.75	7.90
*L-*amphetamine	2.60	4.47	6.63	13.33	1.11	1.79	9.38	15.71	1.14	1.57	−3.13	0.31	1.46	2.33	−5.16	0.02
*D-*methamphetamine	1.28	4.42	−1.56	5.73	1.67	2.66	−3.19	4.96	1.75	2.21	−4.28	4.24	1.67	2.74	7.59	17.89
*L-*methamphetamine	1.66	5.19	−2.83	7.65	2.59	3.56	−3.14	6.69	0.55	0.99	−4.74	7.49	2.17	5.87	8.68	17.91

^*∗*^Indicates combined *D-* and *L-*.

**Table 7:** Dilution study: percent difference from expected with a 4000 (2000) ng/mL standard diluted as indicated. All analytes are based at a 1 : 10 dilution.

Drug/Metabolite	1 : 5 dilution	1 : 10 dilution	1 : 20 dilution	1 : 50 dilution
Amphetamine^*∗*^	1.03	4.04	9.45	12.00
Methamphetamine^*∗*^	−8.17	−0.32	3.13	0.66
*D-*amphetamine	2.38	5.97	9.87	12.19
*L-*amphetamine	2.56	7.39	10.81	9.60
*D-*methamphetamine	−0.51	−3.58	−1.77	−3.63
*L-*methamphetamine	3.89	1.00	5.46	3.87

^*∗*^Indicates combined *D-* and *L-*. High concentration was 2000 ng/ml for individual *D-* and *L-*isomers.

**Table 8:** Stability testing. QC samples were tested for stability after 3 freeze-thaw cycles. They were also tested overnight at the indicated temperatures. A 3- and 7- day postextraction study were also performed at 2–8°C.

Drug/Metabolite	F/T 3 cycles		Overnight stability (%)			Postpreparation stability		
	QC	% Diff	RT	4°C	−20°C	Init % diff nom	% Diff init day 3	% Diff init day 7
Amphetamine^*∗*^	QC 75	0.69	2.48	4.91	3.63	8.95	−1.33	0.61
	QC 800	6.92	6.21	8.19	0.45	−0.69	0.16	−1.78

Methamphetamine	QC 75	−2.38	1.39	-0.23	0.72	−1.25	2.61	6.13
	QC 1800	1.12	2.62	1.95	4.85	−2.22	−4.26	−1.39

^*∗*^Indicates high QC of 800.

**Table 9:** Matrix effects 10 different lots of oral fluid were fortified with QC material to a concentration of 7.5 ng/mL and the %CV determined of the analyte/IS area ratio.

Drug/Metabolite	Matrix comparison
	%CV Analyte/IS ratio
Amphetamine^*∗*^	3.39
Methamphetamine^*∗*^	4.60
*D-*amphetamine	1.59
*L-*amphetamine	2.35
*D-*methamphetamine	7.54
*L-*methamphetamine	3.09

^*∗*^Indicates combined *D-* and *L-*.

**Table 10:** Concomitant medications: the indicated medications prepared in methanol were spiked into a QC 7.5 standard and measured. The data indicates percent difference from a QC standard spiked with blank methanol at the same volume as the drug standards.

Drug/Metabolite	% diff from MEOH spike					
	Dextromethorphan	Phenylephrine	Diphenhydramine	Salicylic acid	Phentermine	OTC mix
Amphetamine	−0.92	−0.06	−0.84	1.89	−0.06	1.39
Methamphetamine	6.15	3.95	6.10	2.67	−2.29	−1.34
*D*-amphetamine	−0.77	−1.64	−2.00	1.06	−0.94	−3.90
*L*-amphetamine	−1.97	−0.41	2.07	1.30	3.21	−0.54
*D*-methamphetamine	−6.26	5.53	−6.10	−3.81	7.45	−5.96
*L*-methamphetamine	−2.10	−0.24	−0.08	0.71	7.73	−0.12

**Table 11:** Transitions and retention times for *D-*and *L-*amphetamine and methamphetamine.

Drug	RT (min)	Q1	Q3	DP	EP	CE	CXP
*D*-amphetamine (S)	6.50	136	91.000	35	10	25	6
*L*-amphetamine (R)	7.21	136	91.101	35	10	25	6
*D*-amphetamine *D*-11	6.51	147.2	98.100	35	10	26	7
*L*-amphetamine *D*-11	7.22	147.2	98.101	35	10	26	7

*D*-methamphetamine (S)	7.91	150	119.100	10	10	25	9
*L*-methamphetamine R	8.45	150	119.101	10	10	25	9
*D*-methamphetamine *D*-11	7.91	161	97.100	85	10	26	7
*L*-methamphetamine *D*-11	8.46	161	97.101	85	10	26	7

*Note.* Retention times are updated using the internal standard each time that the data are analyzed.

## Data Availability

https://doi.org/10.7910/DVN/CQTWE1, Harvard Dataverse.
